# Points

**Published:** 1898-05

**Authors:** 


### POINTS.

Dr. Cooper Curtice, of Moravia, commenting upon the examination of the Gray herd of tuberculous cattle at Elmira, points out the source of infection of the young animals, as indicated by the location and condition of development of the lesions, as arising in a large proportion from the food which, for six months, was skim-milk.

The meat-inspection service of the Department of Agriculture promises to more than exhaust this year the increased appropriation for microscopical inspection of meats, owing to the greatly enhanced demand for these products in foreign markets.

France now imposes a duty of forty dollars on all horses imported into that country. Evidently too many American-bred horses were finding purchasers there.

Dr. John Wende, of Buffalo, has brought forth a very useful and ingenious device of aiding more ready catheterization of the bovine species. The hollow tube is made of sheet-copper, about eight inches long, with a long bevel on one end and square at the other. The bevelled end is introduced through the neck of the bladder, and the ordinary catheter inserted readily through the tube, and after the introduction of the catheter the tube is withdrawn by sliding it out over the catheter.

Asses will be used by the Bureau of Animal Industry in preparing the hog-cholera serum.

The Supreme Court of Iowa recently decided that dogs were chattels, and reversed a decision of a lower court discharging a man for stealing a dog on the grounds that dogs were not property.

The health department of the City of New York is producing tuberculin and mallein. They have nine horses for the production of diphtheria-antitoxin, three for tetanus-antitoxin, three for pneumococcus-antitoxin, two for typhoid-antitoxin, and three for streptococcus-antitoxin.

Veterinarian Langdon, of North Dakota, reports through the local journals successful indications of a curative character in a series of experiments with glandered horses, and is hopeful of finding a reliable cure for this disease. Rather treacherous grounds to be working on, considering the nature of the disease.

The Jury-bill exempting veterinarians of New York and Kings Counties has been signed by the Governor and is now a law.

The Spanish Government is said to have purchased 8000 horses and mules in this country since the trouble in Cuba began.

The United States Government has recently been a large purchaser of horses in Western horse-marts. One order for 2000 was placed at one time.

Veterinarian H. B. Ambler, of Chatham, killed twenty-six tuberculous swine at Nivenville, comprising almost the entire drove. The origin was thought to be from the consumption of offal of tuberculous cattle.

At the annual banquet of the Kansas City Veterinary College, early in March, the entire menu served, from soup to filet, was taken from the choice cuts of horseflesh. It was much enjoyed by all present.

The Examining Board of Horseshoers visited Rochester, Albany, and New York City in April, conducting examinations of unlicensed shoers, veterinary surgeons conducting shoeing shops. The names of all those not complying with the requirements of the law will be placed in the hands of the grand jury for prosecution in the respective districts.

Dr. S. D. Brimhall, field veterinarian to the Minnesota State Board of Health, has recently investigated a series of interesting calk-wounds, carbuncles of the coronary band, etc., among horses in the logging-camps. It had been supposed by local authorities that a new and very infectious disease had appeared, and considerable excitement prevailed.

The New York State Board of Health examined sixty-eight cattle in March, four of which were condemned as tuberculous. In the past nine months five hundred and eight animals were examined, eighty-one of which were condemned and slaughtered.

New Jersey's outgoing Legislature granted $15,000 to the State Board of Health; $12,000 to the Dairy Commission; $5000 to the Tuberculosis Commission; $15,500 to the Agricultural Experimental Station.

The Tuberculosis Committee of the New York State Board of Health continues to examine a small number of cattle each month for tuberculosis.

Dr. E. S. Moyer, of Blooming Glen, Pa., is just recovering from an attack of typhoid fever, after an illness of three months.

